# Demonstration of Integrated Quasi-Vertical DMOS Compatible with the Bipolar-CMOS-DMOS Process Achieving Ultralow R_ON,sp_

**DOI:** 10.3390/nano15030172

**Published:** 2025-01-23

**Authors:** Feng Lin, Tuanzhuang Wu, Weidong Wang, Zhengxuan Wang, Yi Zhang, Sheng Li, Ran Ye, Long Zhang, Jiaxing Wei, Siyang Liu, Weifeng Sun

**Affiliations:** 1 National ASIC System Engineering Research Center, School of Integrated Circuits, Southeast University, Nanjing 210096, China; 230219353@seu.edu.cn (F.L.); 230228400@seu.edu.cn (T.W.); 220226200@seu.edu.cn (W.W.); 220236426@seu.edu.cn (Z.W.); seulisheng@seu.edu.cn (S.L.); 101300309@seu.edu.cn (R.Y.); longzhang@seu.edu.cn (L.Z.); liusy2017@seu.edu.cn (S.L.); 2 CSMC Technologies Corporation, Wuxi 214000, China; zhangyi656@csmc.crmicro.com

**Keywords:** quasi-vertical structure, DMOS, SGT, BCD process, specific ON-state resistance

## Abstract

An integrated quasi-vertical double-diffused MOSFET (DMOS) with split-gate trench (SGT) structure (SGT-QVDMOS), whose specific ON-state resistance (R_ON,sp_) breaks the traditional Si limit significantly, is proposed and fabricated. The measured data of the latest manufactured device is presented. By introducing the vertical gate poly, the split grounded source poly, and the asymmetric thick oxide in the gate trench, the traditional lateral drift region is folded in the SGT-QVDMOS. In this way, the device voltage withstanding mode transforms from one dimension to two dimensions, including the horizontal and the vertical directions. Combining the electric field modulation effect and the reduced lateral area, which benefit from the quasi-vertical structure, the forward conducting characteristic of the SGT-QVDMOS is effectively improved. According to the measured results from the SGT-QVDMOS manufactured by the 180 nm Bipolar-CMOS-DMOS (BCD) process, the ultralow ON-state resistance is obtained. The device achieves 1.9 V V_TH_, 11.07 mΩ∙mm^2^ R_ON,sp_, and 48.6 V BV, which is 39.0% lower than the traditional Si limit.

## 1. Introduction

Owing to the advantages of fast switching, simple driving, easily being isolated in integrated circuits (ICs), and compatibility with Bipolar-CMOS-DMOS (BCD) technology, lateral double-diffused MOSFETs (LDMOS) are the most common power devices adopted by power conversion circuits [1,2,3]. However, for the blocking voltage (BV), which is proportional to the length of the drift region, the conducting and the blocking characteristics of the traditional LDMOS devices are mainly decided by the relatively large lateral size, limiting the reduction in the specific ON-state resistance (R_ON,sp_). Therefore, wide attention has been paid to optimizing the tradeoff between the BV and the R_ON,sp_ of LDMOS, trying to achieve supreme conducting characteristics [4,5,6,7,8].

On the other hand, for discrete devices like vertical double-diffused MOSFETs (VDMOS), the drift region is located in the vertical direction, making the BV independent of the chip area [9]. The current density is then increased obviously, providing an idea to improve the R_ON,sp_ of LDMOS. Technologies including the dielectric trench or field oxide in the drift region [10,11,12,13], the multi-channel trench structure [14,15], the vertical trench [16], and the step split gate [17] have been promoted and investigated. All of them verticalize the part or the entire lateral drift region, aiming to break the limit of the R_ON,sp_ for LDMOS.

To find a novel solution to further improve the conducting performances of the power devices in integrated circuits, a quasi-vertical DMOS with a split-gate trench (SGT) structure (SGT-QVDMOS), which combines the benefits of both LDMOS and VDMOS, has been preliminarily proposed [18]. In this work, the conducting and blocking characteristics of the device are comprehensively analyzed. Demonstrated by the simulation and the latest experiment results, the SGT-QVDMOS with ultralow R_ON,sp_ is achieved.

## 2. Device Structure and Working Mechanism

### 2.1. Device Structure

**Figure 1:**
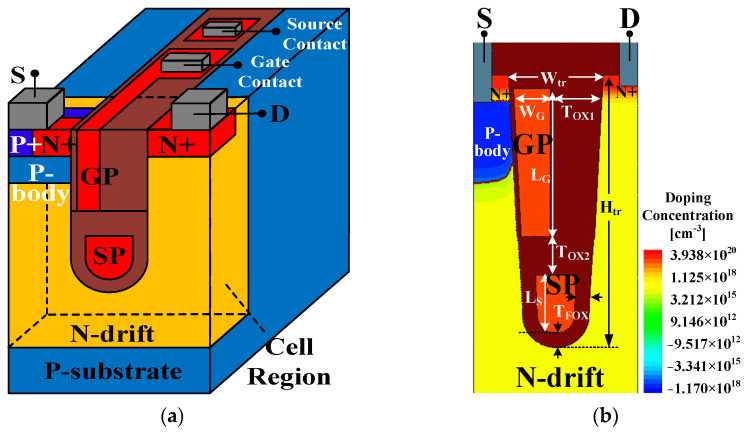
Schematic diagrams of the SGT-QVDMOS. (**a**) The 3D diagram of the entire device and (**b**) the cross-section of the cell structure.

The schematic diagram of the SGT-QVDMOS is presented in Figure 1a. Compared with the 120 V rating left-right SGT structure in [16], the device proposed here adopts an up–down SGT structure. This is because the cell pitch of the up–down SGT structure has more advantages in miniaturization. Therefore, SGT MOSFETs with tens of voltage usually adopt the up–down structure. As shown in Figure 1a, a narrow and deep gate trench is introduced to fold the traditional lateral N-drift region, forming the basic quasi-vertical structure of this device. The source and the drain are on the surface, located on each side of the gate trench. Figure 1b shows the cross-section of the cell region, which is designed to be asymmetric. On the source side, a vertical gate poly (GP) is introduced, which controls the P-body to form the channel. The thickness of the gate oxide (T_GOX_) here is set to be 140 Å. On the other side, to sustain the high gate–drain electric field, the oxide is relatively thick. Moreover, a split gate is adopted, which is the grounded source poly (SP) at the bottom of the trench. The SP and the drift region form a MOS-like structure, providing charge balance under the blocking state, and assisting the expansion of the depletion layer in the drift region. The SP also works as a field plate, which enhances the electric field modulation effect, introducing new electric field peaks in the drift region to increase the BV. The pitch size (W) of the cell in Figure 1b is 0.9 μm, while the mesa width (W_TO_) is 0.4 μm.

In a BCD process, which is a mainstream semiconductor process combining Bipolar (for analog parts), COMS (for logic parts), and DMOS (for power parts) fabrication flows suitable for power integrated circuits, the SGT-QVDMOS is mainly manufactured in a high voltage N-well. The contacts of the gate and the SP are in the P-epi layer, just as shown in Figure 1a.

The critical dimensions are presented in Figure 1b. W_tr_ is the width of the trench, while W_G_ is the thickness of the GP. T_XO1_, T_OX2_, and T_FOX_ are the thicknesses of the oxide layers on the drain side, between the gate and the SP, at the trench bottom, respectively. L_G_, L_S_, and H_tr_ are the length of the GP, the length of the SP, and the depth of the gate trench.

All the dimensions above and the doping concentrations of the P-body (N_pb_), the N-drift (N_dr_), and the N+ region (N_n+_) are listed in Table 1. Apparently, all these parameters need to be carefully designed to obtain an excellent tradeoff between the BV and the R_ON,sp_. Theoretically, a too-thick W_G_ makes the T_OX1_ thinner, affecting the electric field near the drain and decreasing the BV. However, the W_G_ cannot be too thin to make the BV degrade as well. Meanwhile, the R_ON,sp_ decreases when the W_G_ becomes thicker since the GP accumulates electrons near the trench surface on the drain side. Because the L_G_ determines the length of the channel, a large L_G_ will lead to an elevated R_ON,sp_. At the same time, the charge balance effect deteriorates when the L_S_ is too small, resulting in a significant decrease in the BV and an increase in the R_ON,sp_. The T_FOX_ has negligible impact on the R_ON,sp_, as it is very thin. However, when it is too thin, the BV also decreases. Increasing the H_tr_ lengthens the conduction path of the device, thereby increasing its R_ON,sp_. The W_TO_ is a key parameter, which seriously affects the device performance. When it is too small, the narrow current path increases the R_ON,sp_. When it is too large, the drift region cannot be completely depleted, hence decreasing the BV. By increasing the N_dr_, the resistivity decreases, leading to a drop in the R_ON,sp_. However, the RESURF effect of the SP also becomes weak, leading to the degradation in the BV. Moreover, the increase in N_pb_ raises the V_TH_, increasing the R_ON,sp_, while the BV is not influenced as long as the N_pb_ is high enough.

Based on the analysis above, all the critical parameters have been well designed in the preliminary work [18]. The optimized parameters are listed in Table 1, which are adopted to simulate and fabricate the SGT-QVDMOS.

### 2.2. Conducting and Blocking Mechanism

**Figure 2:**
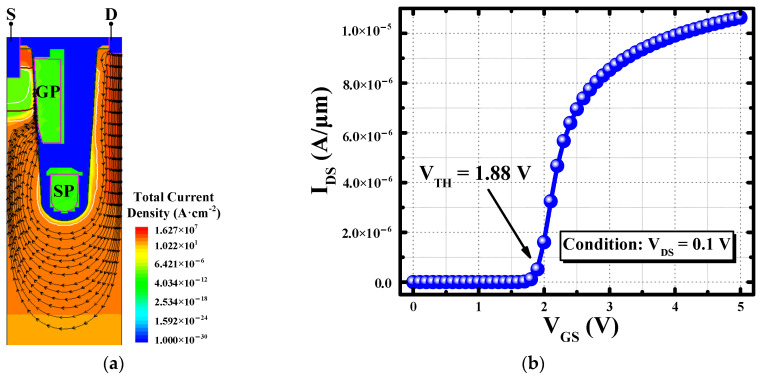
Simulated (**a**) distribution of forward current path in the SGT-QVDMOS, and (**b**) the transfer characteristic of the device.

With the help of Sentaurus TCAD, the conducting and blocking characteristics are analyzed. When the SGT-QVDMOS is ON, the electrons flow from the source, along the side wall of the trench, down to the bottom, and then up to the drain port. It generates the current path shown in Figure 2a. The transfer characteristic of the device is simulated and plotted in Figure 2b. The threshold voltage (V_TH_) is extracted to be 1.88 V. Under V_GS_ = 5 V and V_DS_ = 0.1 V condition, the simulated I_DS_ is 10.63 μA/μm, equaling an R_ON,sp_ of 8.46 mΩ∙mm^2^.

**Figure 3:**
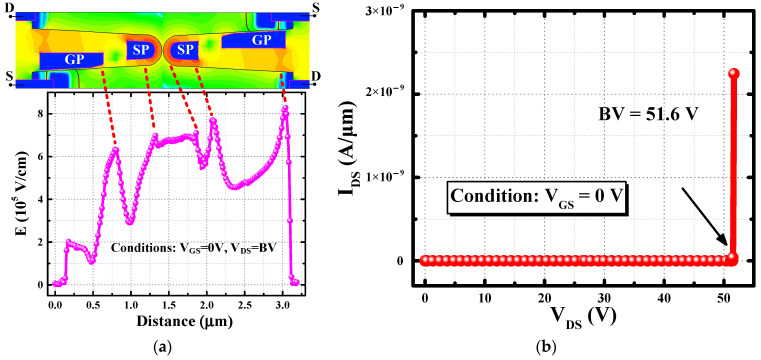
Simulated (**a**) distribution of the electric field along the oxide interface under the breakdown state with V_DS_ = BV, and (**b**) the blocking characteristic of the SGT-QVDMOS.

Figure 3a shows the distribution of the electric field in the SGT-QVDMOS under the breakdown state. The peak electric field (E_peak_) appears in five areas, namely the left bottom corner of the GP, two sides of the bottom corner of the trench, the top right corner of the SP, and the area below the drain electrode. After careful simulation, the five electric field peaks are basically the same. It achieves a similar effect to the super junction structure, which can obviously improve the tradeoff between the BV and the R_ON,sp_. At this time, the withstanding voltage of the device reaches the maximum. The simulated blocking characteristic is shown in Figure 3b. The BV of the device is 51.6 V. It significantly breaks the traditional Si limit [19] by reducing 58.6% of the R_ON,sp_, as shown in Figure 7. By adjusting the parameters in Table 1, the SGT-QVDMOS structure can also be applied in other BV classes. The theoretical results are marked in Figure 7.

## 3. Fabrication and Result Discussions

### 3.1. Process and Fabrication

**Figure 4:**
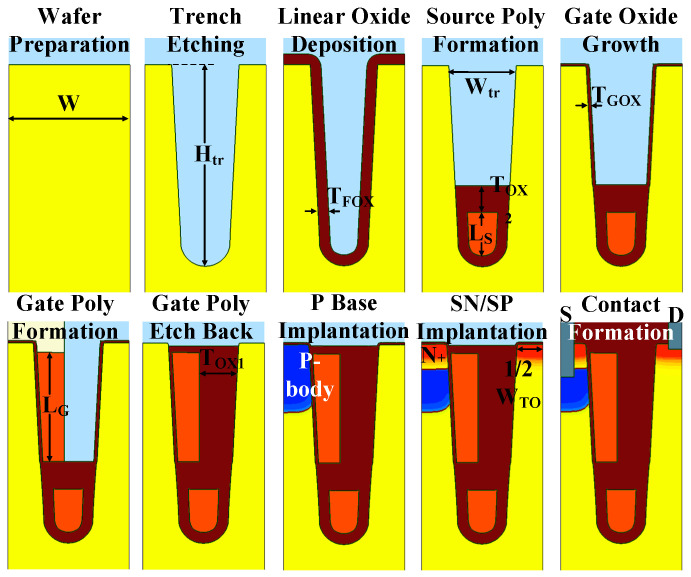
The key process flow of the SGT-QVDMOS based on the 180 nm BCD technology.

The proposed SGT-QVDMOS is manufactured by a 180 nm BCD process. The key process flow is shown in Figure 4. During wafer preparation, an implanted N-well is formed on a P-type substrate. A 1.5 μm trench is formed by reactive ion etching technology, followed by the deposition of an oxide layer in the trench. The N-type polysilicon is then deposited into the trench and is etched back to form the SP. Prior to the formation of the gate oxide, the former deposited oxide needs to be removed. A gate oxide layer is formed by sacrificial oxidation technique and hot oxygen growth. Another poly layer is then deposited and etched back to form the asymmetric GP. Then, the P-body, the N+, and the P+ regions are implanted. Finally, the silicide and the aluminum are deposited to form the Ohmic contact.

**Figure 5:**
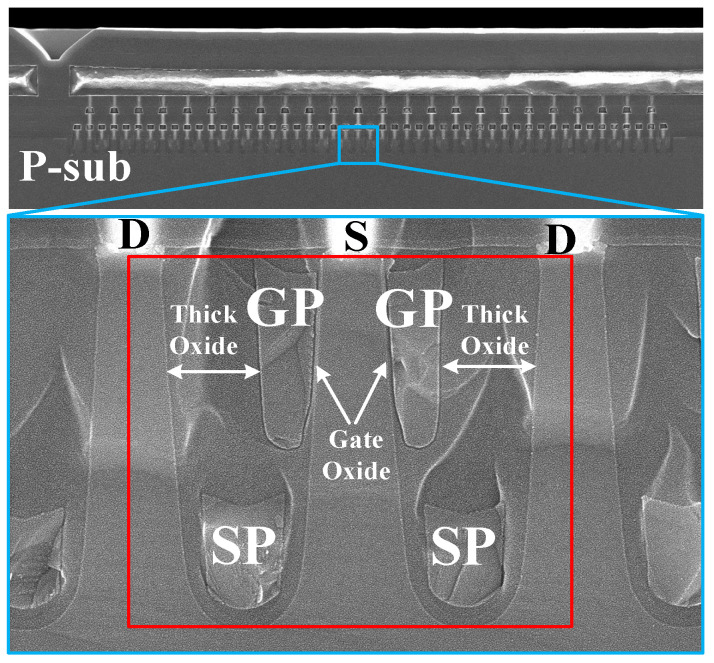
SEM photograph of the manufactured SGT-QVDMOS, whose cell structure is enlarged in the red square.

The SEM photograph of the manufactured SGT-QVDMOS is shown in Figure 5. Clearly, the main structures of the STG-QVDMOS, including the gate trench, the vertical GP, the split SP, and the thick oxide on the drain side, are all well made. What should be noted is that compared with the 250 Å gate oxide in [18], the gate oxide here is reduced to 140 Å to achieve a lower V_TH_ (from 3.7 V down to 1.9 V) and a stronger conducting capability under V_GS_ = 5 V, which is a normal driving condition for an integrated DMOS.

### 3.2. Measurement Results and Discussions

**Figure 6:**
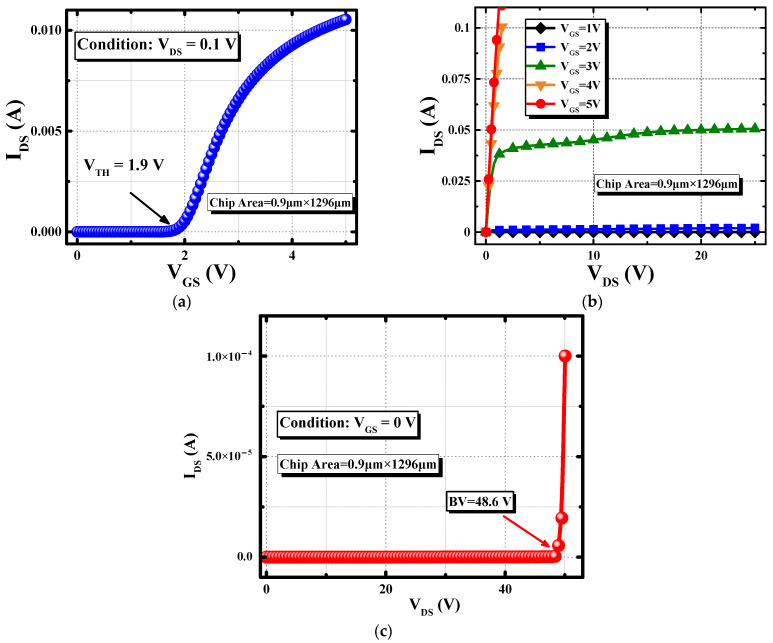
Measured (**a**) transfer, (**b**) conducting, and (**c**) blocking characteristics of the SGT-QVDMOS.

The measured curves of the transfer, the conducting, and the blocking characteristics of the device are plotted in Figure 6. The chip area of each test key is 0.9 × 1296 mm^2^. As shown in Figure 6a, a V_TH_ of 1.9 V is achieved, ensuring a normal conducting state under the V_GS_ = 5 V condition, which can be proved by the I_D_-V_D_ curve in Figure 6b. Unlike the conducting characteristic in [18], the current increment under 5 V gate-source bias becomes saturated, implying that the channel is fully inversed now. Meanwhile, a BV of 48.6 V is obtained, just as presented in Figure 6c, indicating that the SGT-QVDMOS maintains a good blocking property. The measured results of a manufactured device are consistent with the designed values. It demonstrates that the design and the fabrication in this work match well.

**Figure 7:**
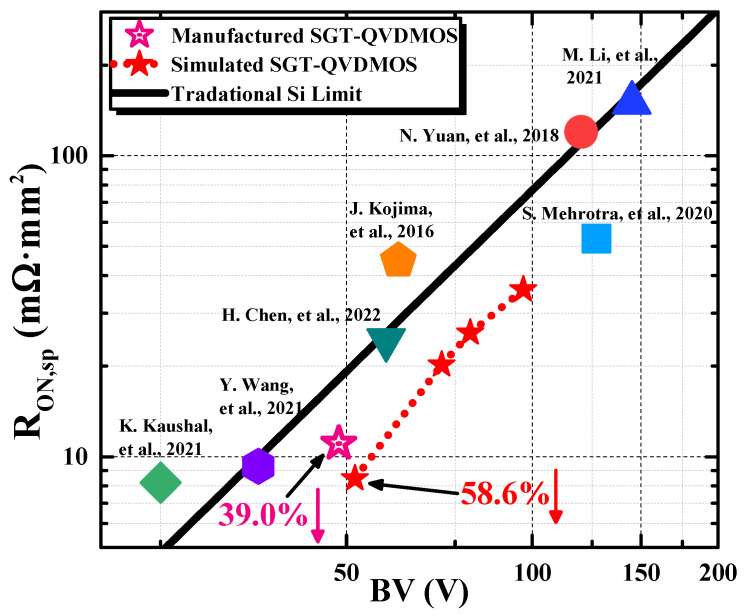
Comparison among the SGT-QVDMOS in this work and other reported milestones, considering the performances of R_ON,sp_ and BV [16,20,21,22,23,24,25].

Extracted from the I_DS_-V_GS_ curve under V_GS_ = 5 V and V_DS_ = 0.1 V condition, the R_ON,sp_ of the device is 11.07 mΩ∙mm^2^. Taking the 48.6 V BV into consideration, the manufactured SGT-QVDMOS breaks the traditional Si limit significantly. A 39.0% reduction in the R_ON,sp_ is achieved, as presented in Figure 7. Typical milestones reported by other researchers are also presented as comparisons [16,20,21,22,23,24,25].

It demonstrates the correctness and the practicality of the SGT-QVDMOS structure. On the one hand, the device has an optimal BV and R_ON_,sp tradeoff that is comparable to discrete SGT-MOS devices. On the other hand, the preparation of the device is compatible with the 0.18 µm BCD process and thereby has the advantage of integration. In general, the device overcomes the disadvantages of discrete devices and integrated devices and combines the advantages of both. It has an advantageous performance in the same type of devices under the identical voltage rating.

## 4. Conclusions

An integrated SGT-QVDMOS device compatible with the BCD process is designed and fabricated in this work. The quasi-vertical structure, which is realized by the asymmetric gate trench and the vertical GP, modifies the drift region from the horizontal direction to the vertical direction, improving the conducting characteristic. Moreover, the split poly at the bottom of the gate trench is adopted. It helps modify the depletion layer in the drift region, contributing to the reduction in the R_ON_. The manufactured SGT-QVDMOS expresses a V_TH_ of 1.9 V and a BV of 48.6 V. Taking the 11.07 mΩ∙mm^2^ R_ON,sp_ under V_GS_ = 5 V and V_DS_ = 0.1 V into consideration, it breaks the traditional Si limit significantly, achieving a 39.0% R_ON,sp_ reduction. The advantages of the SGT-QVDMOS structure, finally, are demonstrated. It is a promising integrated power device solution to further improve the integration and power density of power ICs. In order to industrialize the SGT-QVDMOS and the corresponding BCD process platform, more efforts are expected to be put into investigating the system-level performance and improving the reliability of this device in the near future.

## Figures and Tables

**Table 1:** Key parameters of the SGT-QVDMOS.

Parameters	Optimized Value	Parameters	Optimized Value
H_tr_	1.5 μm	T_OX2_	0.2 μm
W_tr_	0.5 μm	T_FOX_	0.08 μm
L_G_	0.9 μm	W_TO_	0.4 μm
W_G_	0.2 μm	W	0.9 μm
L_S_	0.32 μm	N_dr_	1.1 × 10^17^ cm^−3^
T_GOX_	140 Å	N_pb_	1 × 10^17^ cm^−3^
T_OX1_	0.3 μm	N_n+_	3.0 × 10^20^ cm^−3^

## Data Availability

Data are contained within the article.
